# Kounis Syndrome: Bee Sting-Induced Acute Myocardial Infarction

**DOI:** 10.7759/cureus.47507

**Published:** 2023-10-23

**Authors:** Sonali K Borkar, Priyadarshan Hande, Nandkishor J Bankar

**Affiliations:** 1 Community Medicine, Datta Meghe Medical College, Datta Meghe Institute of Higher Education and Research (Deemed to Be University), Nagpur, IND; 2 Cardiology, Vidarbha Institute of Medical Sciences, Nagpur, IND; 3 Microbiology, Jawaharlal Nehru Medical College, Datta Meghe Institute of Higher Education and Research (Deemed to Be University), Wardha, IND

**Keywords:** coronary arteries, hypersensitive reaction, myocardial infarction, bee stings, kounis syndrome

## Abstract

Acute coronary syndrome or ST-elevation myocardial infarction that develops as a hypersensitive reaction following exposure to an allergen, such as chemicals or bee or wasp stings, is known as Kounis syndrome (KS). Based on angiographic characteristics, three kinds of KS have been identified. Multiple bee stings typically result in localized allergic reactions and anaphylaxis, but they can also occasionally induce severe systemic toxic reactions. Here, a case of KS in a 50-year-old male presented with swelling on the face and upper limbs and breathing difficulties resulting from bee stings which led to myocardial infarction. The risk of KS should be considered by the physician at the primary level in all situations involving multiple bee bites.

## Introduction

Kounis syndrome (KS) is a hypersensitive coronary disorder brought on by a variety of illnesses, medications, environmental exposures, diets, and coronary stents [1]. In KS, acute coronary syndrome (ACS) or ST-elevation myocardial infarction (STEMI) develops as a hypersensitive reaction following exposure to allergens like chemicals or insect stings [2]. Histamine, arachidonic acid derivatives, platelet-activating factor, neutral proteases, and a range of chemokines and cytokines generated during the allergic activation process are some of the inflammatory mediators responsible for this syndrome [3]. Various causes are attributed as triggering factors for KS. These include various drugs such as aspirin, ampicillin, heparin, and diclofenac; conditions such as angioedema, exercise-induced anaphylaxis, and bronchial asthma; consumption of foodstuffs such as canned food, fish, and mushrooms; environmental exposures such as stings of jellyfish, scorpion, honeybees, cobra venom, and viper venom [1]. The sting venom of honey bees, also known as apitoxin, can cause allergic, neurotoxic, and cardiovascular effects. The quantity of venom administered and the number of bites are both related to the severity of systemic reactions [4]. Here, a case of KS is presented with swelling on the face and upper limbs and breathing difficulties resulting from bee stings. The purpose of reporting this instance is to raise awareness of this uncommon syndrome.

## Case presentation

**Figure 1:**
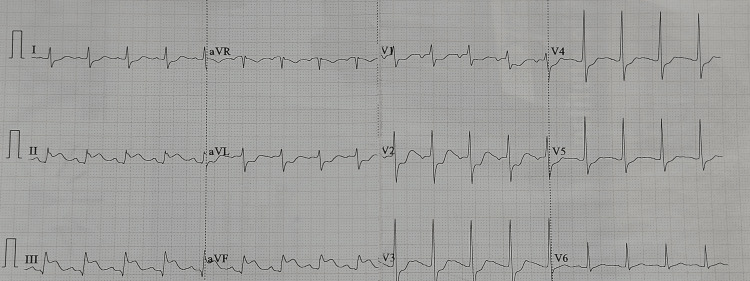
First ECG showing changes suggestive of inferior and posterior wall myocardial infarction

**Figure 2:**
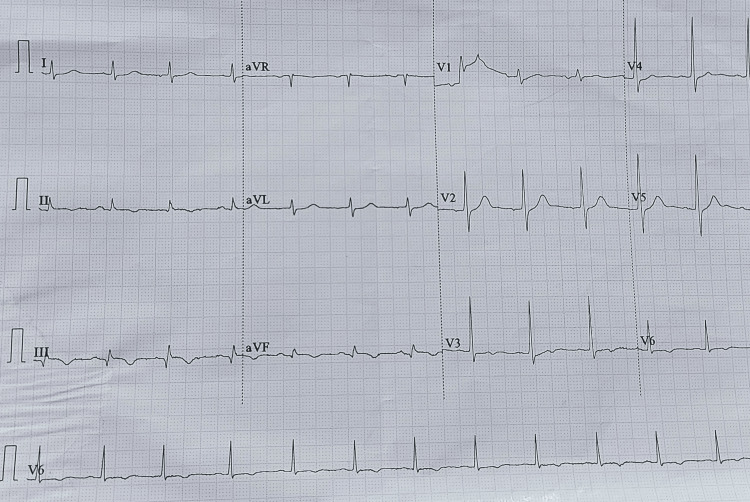
Follow-up ECG showing regression of ST-segment changes

A 50-year-old male patient reported to casualty of the peripheral center with a history of 100-150 honeybee bites. He was having facial puffiness with swelling on both the upper limbs and difficulty in breathing. On examination, the patient was drowsy with bradycardia and hypotension. He was intubated immediately and respiratory support was given. The patient immediately received steroids, adrenaline, and antihistaminic. The patient became conscious 30 minutes later and mentioned experiencing chest pain. An electrocardiogram (ECG) suggested inferior and posterior wall myocardial infarction (Figure 1). He was given antiplatelets (Tab. aspirin 325 mg and Tab. clopidogrel 300 mg), Tab. rosuvastatin 40 mg, and low molecular weight heparin (Inj. enoxaparin 60 mg subcutaneous). Follow-up ECG after six hours and the next day morning suggested regression of ST-segment changes (Figure 2). The patient was referred to the tertiary care center for further management.

**Figure 3:**
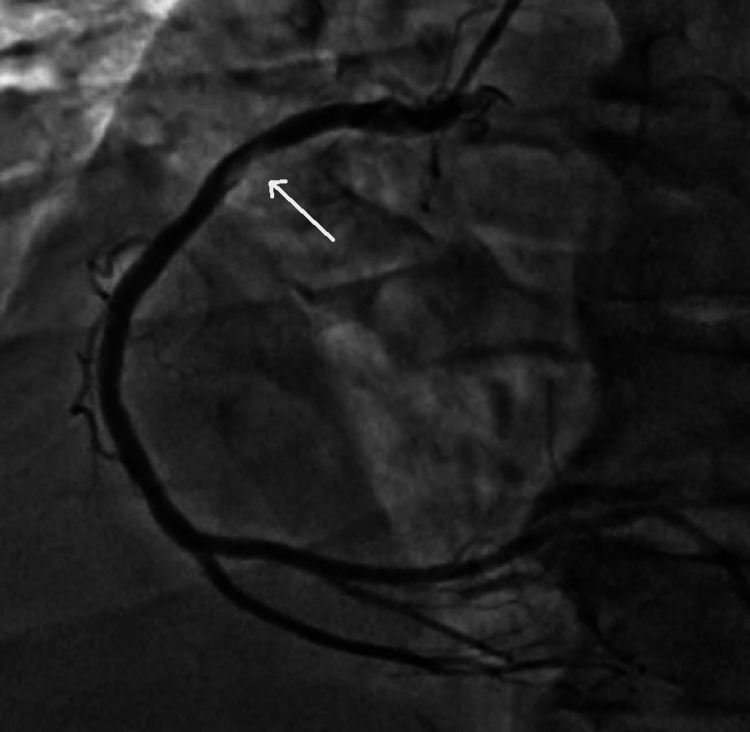
Angiography showing right coronary artery occlusion (80%)

In the tertiary care center, the patient was weaned off from the ventilator on the second day. Antiplatelet (Tab. aspirin 75 mg and Tab. clopidogrel 75 mg) once daily and anticoagulant therapy (Inj. enoxaparin 60 mg subcutaneous) twice daily was given during the hospital stay. The patient underwent coronary angiography on the fourth day. Angiography was suggestive of 80% thrombotic proximal right coronary artery occlusion and 80% stenosis in the left circumflex artery, for which coronary angioplasty was advised (Figure 3). The patient was kept on antiplatelet and anticoagulation therapy (Tab. aspirin 75 mg and Tab. acitrom 2 mg once daily with international normalized ratio in the range of 2.0-3.0).

**Figure 4:**
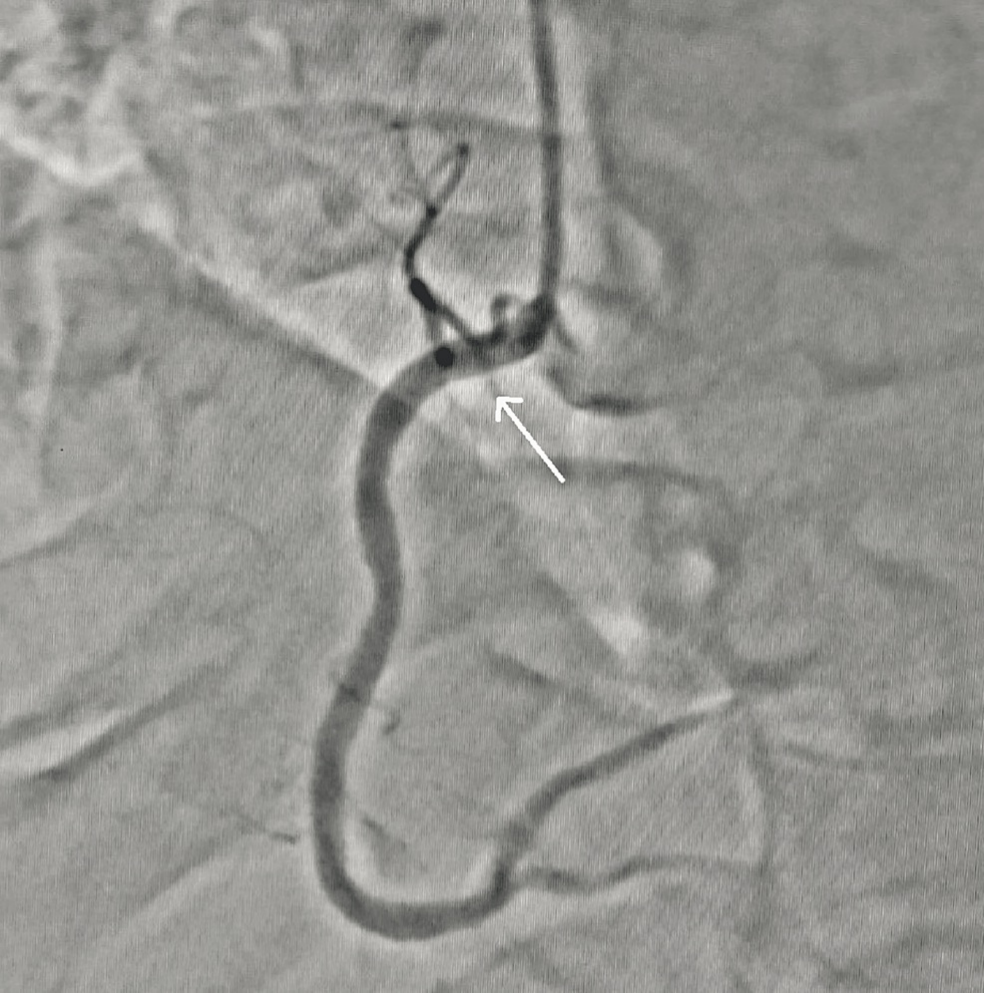
Check angiography suggestive of the resolution of the right coronary artery thrombus

Check angiography was done after 15 days of discharge which was suggestive of the resolution of the right coronary artery thrombus (Figure 4). The patient underwent percutaneous transluminal coronary angioplasty to the left circumflex artery on the same day. The patient was advised to take antiplatelets and statin therapy (Tab. aspirin 75 mg, Tab. clopidogrel 75 mg, and Tab. rosuvastatin 20 mg once daily). The patient was doing well during a one-month follow-up visit.

## Discussion

In India, especially in rural areas, honey bee stings are frequent, particularly in communities where beehives are frequently found. More than 50 bee stings at once signify massive honey bee envenomation [4]. Envenomation caused by bee stings can range from minor allergic reactions to life-threatening anaphylaxis [5]. The intensity of systemic reactions is correlated with the amount of venom injected and the number of bites. Acute kidney injury, acute myocardial infarction (AMI), stroke, immune thrombocytopenia, and multiorgan failure syndrome are examples of severe systemic consequences [4].

The prevalence of KS among patients hospitalized for allergy, hypersensitivity, and anaphylactic reactions was 1.1% overall (unstable angina, 0.2%; STEMI, 0.1%), according to the first nationwide epidemiological study on KS conducted in the United States of America [6]. According to Kounis in 1991, "allergic angina" is a term for chest pain that simultaneously develops with allergic reactions, as well as clinical and biochemical signs of classic angina pectoris brought on by inflammatory mediators produced during the allergic insult. The term "allergic MI" refers to the development of allergic angina into AMI [2]. Examples include reactions to a variety of pharmaceuticals (such as antibiotics, anticancer drugs, and nonsteroidal antiinflammatory drugs), contrast exposure, poison ivy, bee stings, and shellfish. The term "Kounis syndrome" now includes coronary arterial involvement in addition to other arterial systems with comparable physiologies, such as the mesenteric and cerebral circulation, which leads to ischemia/infarction of the supplied important organs [7]. There are two suggested mechanisms; however, the actual underlying mechanism is not fully understood. KS is linked to allergic, hypersensitive, anaphylactic, and anaphylactoid reactions [8]. Based on angiographic characteristics, three kinds of KS have been identified. The type I variant includes patients with unstable angina, which can develop into AMI and is brought on by coronary artery spasm in a previously healthy artery. This variant may be a symptom of microvascular angina or endothelial dysfunction. Patients with the type II variant are those in which an acute allergy episode causes a plaque erosion or rupture on a dormant prior atheromatous illness appearing as an AMI. The type III variant includes patients with coronary stent thrombosis (subtype A) or stent restenosis (subtype B) brought on by allergic inflammation [2]. The present case belongs to the type II variant of KS.

ACS following bee sting is uncommon, commonly misdiagnosed, and infrequently recorded [2]. Recent studies have demonstrated that this disease can occur in people of any race, age, or region (from 2 to 90 years old). Ages 40 to 70 make up the majority of those affected (68%) [9]. In recent years, similar cases were reported by Sunder et al., Pirasath et al., and Thwe et al. [5,10,11]. Kadeli et al. reported a case of a severe allergic response to the angiography dye, which led to an inferior wall myocardial infarction [7]. Gopinath et al. reported a case of bee sting-induced KS with atrial fibrillation [12]. Prasad et al. reported a case of multiorgan dysfunction following multiple bee stings [4]. Other cases were reported of KS resulting from various allergens such as cobra bite, centipede bite, oral aspirin, and salad [13-16].

## Conclusions

AMI is one of the most severe and difficult cardiovascular consequences caused by KS. ACS following bee stings is uncommon, commonly misdiagnosed, and infrequently recorded. The risk of KS should be considered by the physician at the primary level in all situations involving multiple bee bites. Awareness should be raised regarding the seriousness of multiple bee stings-induced ACS in general physicians and healthcare professionals working at the primary level. This can be accomplished locally by conducting seminars and providing health information via the media.

## References

[1] Kounis NG (2016). Kounis syndrome: an update on epidemiology, pathogenesis, diagnosis and therapeutic management. Clin Chem Lab Med.

[2] Rao ST, Basappa H, Raveesh H, Hegde S, Manjunath CN (2021). Acute coronary syndrome following honey bee sting: a series of 6 cases of “Kounis syndrome” with literature review. J Indian coll cardiol.

[3] Kounis NG (2013). Coronary hypersensitivity disorder: the Kounis syndrome. Clin Ther.

[4] Prasad SK, Mehta SK, Satyanarayan B, Panda SK (2020). Multi-organ dysfunction following honeybee bite - a rare entity. J Family Med Prim Care.

[5] Pirasath S, Senthan V, Seneviratne MH (2021). Kounis syndrome: acute myocardial infarction following multiple bee stings. SAGE Open Med Case Rep.

[6] Kounis NG, Koniari I, Velissaris D, Tzanis G, Hahalis G (2019). Kounis syndrome - not a single-organ arterial disorder but a multisystem and multidisciplinary disease. Balkan Med J.

[7] Kadeli D, Mangesh D, Keshava R, Gopi A (2019). Kounis syndrome: allergic myocardial infarction!!. J Indian coll cardiol.

[8] (2023). Kounis syndrome. https://litfl.com/kounis-syndrome.

[9] Li J, Zheng J, Zhou Y, Liu X, Peng W (2018). Acute coronary syndrome secondary to allergic coronary vasospasm (Kounis Syndrome): a case series, follow-up and literature review. BMC Cardiovasc Disord.

[10] Sunder A, Mohanty B, Singh A (2020). Kounis syndrome: a rare case. J Family Med Prim Care.

[11] Thwe EE, Sudnik P, Dobrovolschi C, Krishnamurthy M (2022). Kounis syndrome: an allergic acute coronary syndrome due to a bee sting. Cureus.

[12] Gopinath B, Kumar G, Nayaka R, Ekka M (2022). Kounis syndrome and atrial fibrillation after bee sting: a case report. J Family Med Prim Care.

[13] Priyankara WD, Manoj EM, Gunapala A, Ranaweera AG, Vithanage KS, Sivasubramanium M, Snajeeva E (2019). Cardiogenic shock due to Kounis syndrome following cobra bite. Case Rep Crit Care.

[14] Yildiz A, Biçeroglu S, Yakut N, Bilir C, Akdemir R, Akilli A (2006). Acute myocardial infarction in a young man caused by centipede sting. Emerg Med J.

[15] Hangouche AJ, Lamliki O, Oukerraj L, Dakka T, Doghmi N, Zarzur J, Cherti M (2018). Kounis syndrome induced by oral intake of aspirin: case report and literature review. Pan Afr Med J.

[16] Vaina S, Chrysohoou C, Bonfanti L, Kounis NG, Cervellin G, Georgiopoulos G, Tousoulis D (2020). Anaphylactic cardiovascular collapse manifesting as myocardial infarction following salad consumption. A case of Kounis variant type I syndrome. Acta Biomed.

